# Clinical presentation and outcomes of patients with acute rheumatic fever and rheumatic heart disease seen at a tertiary hospital setting in Port Elizabeth, South Africa

**DOI:** 10.5830/CVJA-2017-019

**Published:** 2017

**Authors:** Zongezile Masonwabe Makrexeni, Lungile Pepeta

**Affiliations:** Paediatric Cardiology Unit, Walter Sisulu University, Port Elizabeth, South Africa; Faculty of Health Sciences, Nelson Mandela Metropolitan University, Port Elizabeth, South Africa

**Keywords:** acute rheumatic fever, rheumatic heart disease, left ventricular dysfunction, rheumatic valve surgery, disease outcomes, prevention

## Abstract

**Background:**

The incidence of acute rheumatic fever (ARF) and rheumatic heart disease (RHD) has waned in Western countries, however that is not the situation in developing nations.

**Methods:**

Records were reviewed of patients from the Eastern Cape municipal districts who presented to the Paediatric Cardiology Unit with ARF and RHD from January 2008 to August 2015.

**Results:**

Total of 56 patients with ARF/RHD was reviewed. The majority of patients (n = 52) presented for the first time with RHD. Four patients presented with ARF and two had recurrent ARF. Six patients presented with a combination of RHD and congenital heart disease. Twenty-three patients were operated on for chronic rheumatic valve disease, with good outcomes.

**Conclusion:**

The true burden of ARF/RHD is unknown in the Eastern Cape. Prospective studies are needed to accurately determine the prevalence of RHD in this province.

## Background

Acute rheumatic fever (ARF) is a post-infectious, non-suppurative sequela of pharyngeal infection with Streptococcus pyogenes or a group A beta-haemolytic streptococcus.^1^ More than one-third of affected children develop carditis, followed by progressive and permanent valvular lesions.^2^ Devastating complications of rheumatic heart disease (RHD) include severe valvular regurgitation, heart failure, strokes and infective endocarditis, usually affecting both younger school-going children and economically active, child-bearing members of society.^3^

Industrialised countries have reported the virtual disappearance of RHD.^4^ The burden of RHD in developed countries declined drastically at the end of the 20th century, largely due to reduced overcrowding and improved sanitation and living conditions.^5^ By contrast, in developing countries, RHD remains a public health issue and is the principal cause of acquired heart disease in children and young adults.^6^-^8^ It is estimated that there are over 15 million cases of RHD worldwide, with 282 000 new cases and 233 000 deaths annually.

There was a noticeable decline in cases of ARF/RHD among children under the age of 14 years in a tertiary care hospital in South Africa over a period of 17 years, from 63 cases in 1993 to three cases in 2010.^3^ It was postulated that the decline was probably due to improved access to medical care for the general population, and the introduction in 1994 of free healthcare to children under the age of six years.

This decline was also demonstrated by Smit and co-workers in 2015 in an echocardiography-based prevalence study of RHD in another tertiary care setting in South Africa,^9^ where the prevalence rate was 4.9/1 000 learners in grades 10 to 12. This was much lower than the 12.2/1 000 clinical prevalence rate reported by McClaren et al. in a similar South African population in 1972.^10^ Smit et al. postulated that socio-economic and rural development in South Africa have initiated this decline in RHD prevalence in South Africa.^9^

Although RHD remains a public health issue in developing countries, it appears that in South Africa there has been a decrease in the prevalence of RHD since 1994, compared with earlier studies. However, we need more inclusive, multicentre, prospective studies to confirm the overall prevalence of RHD in South Africa. We therefore conducted a retrospective cohort study to document the clinical presentation and outcomes of patients with ARF/RHD who presented at a tertiary paediatric cardiology referral centre in the Eastern Cape Province, South Africa.

## Methods

This was a retrospective review of records of paediatric patients presenting with ARF and RHD at Dora Nginza Hospital, Eastern Cape, South Africa, from January 2008 to August 2015 (seven years and eight months). The study was conducted following ethics clearance from the Health Research Ethics and Biosafety committee of Walter Sisulu University and permission from the chief executive officer of Dora Nginza Hospital.

Demographic data such as age, gender, origin and clinical presentation of either ARF or RHD, and disease severity and surgical interventions were recorded. The diagnosis of ARF and RHD was based on clinical and echocardiographic evidence of RHD, using the newly revised Jones criteria of the World Heart Federation of 2012.^11^ In addition, an antistreptolysin O titre (ASOT) was performed in all patients as an indication of recent streptococcal infection.^12^

Disease severity was defined according to the task force on the management of valvular heart disease of the European Society of Cardiology and the ESC committee for practice guidelines.^13^ The newly revised clinical criteria for the diagnosis of ARF/RHD by the World Health Federation 201211 can be seen in Table 1.

**Table 1:** Newly revised clinical criteria for the diagnosis of ARF/RHD by the World Health Federation 2012^11^

*Criteria*	*Symptoms*
*Major criteria for the diagnosis of ARF*	
Low-risk population	Carditis (clinical and or subclinical), arthritis (polyarthritis only), chorea, erythema marginatum, subcutaneous nodules
Moderate-and highrisk population	Carditis (clinical or subclinical), arthritis (including mono-arthritis, polyarthritis or polyarthralgia), chorea, erythema marginatum, subcutaneous nodules
Minor criteria for the diagnosis of ARF	
Low-risk population	Polyarthralgia, fever (≥ 38.5°C), ESR ≥ 60 mm/h and/ or CRP ≥ 3.0 mg/dl, prolonged PR interval on electrocardiogram
Moderate- and highrisk population	Mono-arthralgia, fever (≥ 38°C), ESR ≥ 30 mm/h and/ or CRP ≥ 3.0 mg/dl, prolonged PR interval on electrocardiogram
Criteria for diagnosis of ARF: two major criteria required, or one major criterion with two minor criteria	
Echocardiographic diagnosis of RHD	
Pathological mitral regurgitation	Defined as jet length > 2 cm in at least one view, velocity > 3 m/s for one complete envelope and a pansystolic jet in at least one envelope
Pathological aortic regurgitation	Defined as a jet length ≥ 1 cm in at least one view, velocity > 3 m/s in early diastole and pan-diastolic jet in at least one envelope

ESR, erythrocyte sedimentation rate; CRP, C-reactive protein

## Results

A total of 56 patients with ARF and RHD were seen and/or admitted at the Paediatric Cardiology Unit from January 2008 to August 2015. Ninety-three per cent (n = 52) of patients presented for the first time with RHD (all were new patients). The majority (n = 37) of patients in our cohort were from the OR Tambo district of the former Transkei region of the Eastern Cape.

The average age at presentation is given in Table 2. Ninetythree per cent of patients (n = 52) were between the age of five and 15 years, and the remainder were below the age of five years (n = 4), with the youngest patient three years old.

**Table 2:** Extent of the valvular disease

*Extent of the valvular disease*	*Number (n = 56)*	*Average age at diagnosis (years)*
Isolated MV regurgitation	31	10
MV regurgitation with MV stenosis	7	11
MV regurgitation with AV regurgitation	14	10
AV regurgitation	3	10
AV regurgitation with AV stenosis	1	11

MV, mitral valve; AV, aortic valve.

In terms of clinical disease presentation, most patients (n = 52) presented with chronic RHD. Out of this group, two presented with acute-on-chronic RHD in that, in addition to having echocardiographic evidence of chronic RHD, they had a raised ASOT, fever and raised levels of inflammatory markers (C-reactive protein and erythrocyte sedimentation rate). These patients were aged eight and nine years old. All the patients younger than five years old had ARF diagnosed by the revised Jones criteria.^11^,^12^

Six patients presented with a combination of RHD and congenital heart disease (three atrial septal defects, two patent ductus arteriosus and one ventricular septal defect). The most commonly involved valve was the mitral valve, followed by the aortic valve (Table 2). Of the total cohort, only four patients presented with isolated aortic valve disease.

All patients had assessment of left ventricular function preand post-operatively. Twelve of 23 (52%) patients had dilated left ventricular end-diastolic diameter (LVEDD > 50 mm) pre-operatively. Of those, nine out of 12 (75%) had improved to normal left ventricular end-diastolic diameter (LVEDD < 50 mm) post-operatively (p < 0.05). Six of 23 (26%) patients had left ventricular systolic dysfunction (ejection fraction < 55%) pre-operatively.

The mean follow-up time for patients after surgery was six months. Over the study period 23/56 (41%) patients were operated on for chronic rheumatic valve disease. Twelve patients had mitral valve repair, five mitral valve replacement, three aortic valve repair and three double valve (mitral and aortic valve) replacement. The average age at surgery was 11 years. No patients had surgical valvotomy or balloon valvuloplasty. Four patients required re-operation with mitral valve replacement following failed mitral valve repair.

A single patient with atrial flutter post operatively, who was treated with amiodarone, had good control of the arrhythmias. Only one patient died in this cohort, and the cause of death was non-cardiac related (suicide).

## Discussion

A total of 56 patients were reviewed in this study from 2008 to 2015. Of note, the majority of patients in our cohort (n = 37; 66%) were from the OR Tambo district, Eastern Cape. These patients started attending the cardiology clinic at Dora Nginza Hospital from 2012, and were included in the study from 2012 onwards.

Our results suggest that RHD continues to be a scourge in children from the OR Tambo district, whereas the disease burden seems to be declining elsewhere in South Africa.^3^,^9^ A study done by Cilliers^3^ in the Chris Hani/Baragwanath Hospital from 1993 to 2010 showed a decline in the number of cases of RF/RHD from 64 in 1993 to three in 2010. This decline in numbers is thought to be due to improved access to medical care in South Africa since 1994. Although we did not have a reference study in our population, we do see more patients than was reported by Cilliers.

Smit et al.^9^ also reported a decline in the prevalence of RHD in the preliminary results of the Wheels-of-Hope outreach programme, with a prevalence rate of 4.9/1 000 leaners in grades 10 to 12 in central South Africa. This was postulated to be due to improved rural and socio-economic development in South Africa since 1994.

The majority (93%) of patients in our study were within the expected age range of patients affected by this disease.^1^ However there were patients younger than five years of age (n = 4) in our cohort. This is in line with the report by Tani et al.14 on children younger than five years who presented with ARF and were diagnosed using the revised Jones criteria.^11^ In our study, the median age was four years (range 1.9–4.9 years). This emphasises that even though ARF/RHD commonly presents at between five and 15 years of age, there are cases that present early (under five years) who meet the criteria for the diagnosis of ARF.

Regarding clinical presentation, the majority (n = 52; 93%) of our patients presented for the first time with RHD. Only four patients presented with ARF as their first presentation. This may have be due to cases of ARF being missed at the primary healthcare level or patients not presenting early at the clinic with a sore throat.

In the Soweto study, Sliwa et al.^15^ reported on adult cases who presented for the first time with RHD. No patients presented with ARF. This suggests that patients who are not diagnosed early with ARF may present later with established RHD. As a result, they made the suggestion that new cases of RHD should be notified in order to ensure registration and follow up of these patients. Lack of notification of ARF/RHD may result in underreporting. Nkgudi et al. mentioned under-reporting of ARF in their study^16^ and concluded it was due to poor administration of the ARF notification system.

The majority of patients who underwent valvular surgery in our study had mitral and aortic valve surgery. Cilliers reported this as the commonest surgery for RHD.^2^ Timeous surgery for RHD has very good outcomes.^17^

A limitation of this study is that it was a retrospective chart audit of patients seen and admitted to one hospital. The fact that these patients were mostly referred from other hospitals made it very challenging to estimate the incidence of ARF/RHD, as there was no primary screening of subjects suspected to have ARF/RHD.

Despite the limitations, the findings have important implications. RHD should be notifiable, as the majority of our patients presented with RHD. Prospective studies are required in the Eastern Cape to determine the true prevalence of ARF/RHD.

## Conclusion

The true burden of ARF/RHD in the Eastern Cape Province is unknown and therefore prospective studies are needed to determine the prevalence or incidence of ARF and RHD in this province.
